# Association between COVID-19 Infection or Vaccination Outcomes and Methylenetetrahydrofolate Reductase Gene Polymorphism: A Systematic Review of the Literature

**DOI:** 10.3390/jpm13121687

**Published:** 2023-12-05

**Authors:** Ivana Jukic, Aisling Heffernan, Alisa Franceska Schelling, Visnja Kokic Males, Nora Josipa Savicevic, Vedran Kovacic

**Affiliations:** 1 Internal Medicine Department, Gastroenterology Division, University Hospital of Split, 21000 Split, Croatia; 2 Department of Health Studies, University of Split, 21000 Split, Croatia; kokicvisnja@gmail.com; 3 School of Medicine, University of Split, 21000 Split, Croatia; aisling-heffernan@hotmail.com (A.H.); alisa.schelling@web.de (A.F.S.); norajosipa88@gmail.com (N.J.S.); vedran.kovacic.split@gmail.com (V.K.); 4 Internal Medicine Department, Endocrinology Division, University Hospital of Split, 21000 Split, Croatia; 5 Internal Medicine Department, Division of Emergency and Intensive Medicine with Clinical Pharmacology and Toxicology, University Hospital of Split, 21000 Split, Croatia

**Keywords:** methylenetetrahydrofolate reductase gene polymorphisms, COVID-19 infection, SARS-CoV-2, MTHFR C667T, MTHFR A1298C, vaccine

## Abstract

Background: Thrombosis is a detrimental sequala of COVID-19 infection; thus, prophylactic anti-coagulant therapy has been deemed mandatory in treatment unless serious contraindications are present. Susceptibility to thromboembolic events in COVID-19, or following COVID-19 vaccination, is likely attributable to an interplay of factors, including a patient’s baseline clinical status and comorbidities, alongside genetic risk factors. In Europe, 8–20% of the population are homozygous for the MTHFR (methylene tetrahydrofolate reductase) variant, which compromises folate metabolism and elevates homocysteine levels. While heightened homocysteine levels are considered a risk factor for thromboembolic events, the precise clinical significance remains a contentious issue. However, recent research suggests elevated homocysteine levels may predict the course and severity of COVID-19 infection. Given the lack of reliable biomarkers predictive of COVID-19 thrombotic risk existing in practice, and the accessibility of MTHFR screening, we established two main outcomes for this study: (1) to determine the association between hereditary MTHFR mutations and COVID-19 severity and thromboembolic events and (2) to determine the link between MTHFR variants and adverse thrombotic events following COVID-19 vaccination. Methods: The review was conducted in accordance with PRISMA guidelines. Medline, Scopus, and Web of Science databases were searched from pandemic inception (11 March 2020) to 30 October 2023. Eligibility criteria were applied, and data extraction performed. Results: From 63 citations identified, a total of 14 articles met the full inclusion criteria (8 of which were cross-sectional or observational studies, and 6 were case studies or reports). Among the eight observational and cross-sectional studies evaluating the relationship between MTHFR variants (C667T; A1298C) and thromboembolic events in COVID-19 infection, four studies established a connection (*n* = 2200), while the remaining four studies failed to demonstrate any significant association (*n* = 38). Conclusions: This systematic review demonstrated a possible association between the MTHFR gene variants and COVID-19 severity, thromboembolic events, and adverse events following vaccination. However, the paucity of robust data precluded any firm conclusions being drawn. Further prospective trials are required to determine the connection between the MTHFR gene variant and COVID-19 infection and vaccination outcomes.

## 1. Introduction

Severe acute respiratory syndrome coronavirus 2 (SARS-CoV-2) is a strain of coronavirus that causes COVID-19 [1]. First detected in Wuhan, China, the viral pathogen spread rapidly across the world, resulting in a global pandemic and infecting over 630 million individuals [2]. The clinical manifestation of COVID-19 varies significantly, ranging from asymptomatic illness to flu-like syndromes and death [3]. It was initially believed that shortness of breath and pneumonia were the primary causes of mortality; however, emerging evidence indicates that thromboembolic events may be the most severe manifestations [3]. Elevated D-dimer and fibrinogen, two biomarkers of coagulation, correlate with poorer COVID-19 clinical outcomes [4,5]. The propensity to develop detrimental COVID-19 complications is likely a complex interplay of the patient’s clinical status and comorbidities alongside genetic risk factors [6]. A host of susceptibility genes and genetic variants have been linked to an increased risk of developing severe COVID-19. Of particular interest is the MTHFR gene, encoding for 5,10-methylenetetrahydrofolate reductase, a key regulatory enzyme in folate metabolism [7]. Compromised MTHFR function, resulting from inherited mutations, is the most common cause of genetic hyper-homocysteinemia [8].

It is estimated that 20–40% of White and Hispanic individuals in the United States are heterozygous for the most common mutation, *MTHFR C667T* [9]. Heterozygosity reduces MTHFR enzymatic activity to 65% of normal, and affected individuals display mild elevations in homocysteine levels compared with non-mutated controls [8,9]. In Europe, 8–20% of the population is homozygous for the variant. The presence of these two mutations, further decreases the functional capacity of MTHFR to 30% of normal activity. Thus, homozygotes have more pronounced elevations in homocysteine levels than their heterozygous counterparts [8,9]. Although more limited data are available concerning the *A1298C* variant, a 2015 study estimated a prevalence of 7–12% in North American, European, and Australian populations [9]. Historically, the significance of increased plasma homocysteine has been a contentious issue. However, epidemiological studies have demonstrated an association between plasma homocysteine, arterial thrombosis, and venous thromboembolism [10].

Moreover, recent data suggest increased plasma homocysteine may predict the course and severity of COVID-19 [11,12]. In addition, it has been reported that the presence of *MTHFR* polymorphisms alone, even in absence of perturbed homocysteine levels, may contribute to a prothrombotic milieu [13]. Identifying individuals who are at a greater risk of COVID-19 thrombotic complications is essential to assessing patient risk and guiding appropriate thromboprophylaxis and treatment. Thrombosis is an extremely dangerous complication in elderly patients with COVID-19. Since the first months of the pandemic, anticoagulants have been mandatory in treatment protocols for patients with COVID-19, unless there are serious contraindications.

Despite remarkable research efforts to date, clinicians still lack reliable predictive biomarkers to stratify patients for their risk of COVID-19 progression. Given the accessibility of MTHFR screening and the implications of thrombosis in COVID-19 patients, our review aimed to collate the data pertaining to (1) the association between hereditary MTHFR mutations and COVID-19 severity and thromboembolic events and (2) the link between MTHFR variants and adverse thrombotic events following COVID-19 vaccination.

## 2. Materials and Methods

This study was conducted according to the Preferred Reporting Items for Systematic Reviews and Meta-Analyses (PRISMA) guidelines [14].

### 2.1. Information Source and Search Strategies

A literature search was performed in October 2023 to collate relevant studies addressing the review questions. For the period from pandemic’s inception on 11 March 2020 to 30 October 2023, three electronic databases were searched comprehensively to identify relevant articles by using both free text terms and appropriate indexing terms. The databases were (1) Medline (https://www.ncbi.nlm.nih.gov/pubmed, accessed on 10 October 2023), (2) Scopus (https://www.scopus.com/, accessed on 10 October 2023), and (3) Web of Science Core Collection (https://www.webofscience.com/, accessed on 10 October 2023). For each of the three databases, the basic search strategy employed was as follows: “(COVID-19 OR SARS-CoV-2) AND (MTHFR)”. Furthermore, to minimise the number of irrelevant returns, the search was limited to studies with human subjects and those published in the English language. Additionally, reference lists of the included studies were screened for the identification of potentially relevant literature.

### 2.2. Eligibilty Criteria

Studies in the present systematic review met the following inclusion criteria: (1) original observational research, including cross sectional, cohort, case-series, case-studies and reports; (2) studies evaluating MTHFR genotypes and COVID-19 severity in the context of thromboembolic events; and (3) studies reporting data on MTHFR polymorphisms and thrombotic events after COVID-19 vaccination. Inclusion criteria were as follows: human studies only, an adult or young study population (without an age limit), participants could be of male or female gender, diagnosis of COVID-19 acute infection or COVID-19 vaccination, all MTHFR gene variants in the study population had been determined, and there were thromboembolic events and COVID-19 severity was in the context of these thromboembolic events. Exclusion criteria for the review were as follows: (1) studies with primary endpoints which were outside the scope of our review (e.g., MTHFR polymorphisms or COVID-19-associated neuro–psychiatric conditions), (2) narrative reviews or commentary, (3) animal or in vitro research, (4) studies lacking MTHFR genotypic data, (5) research published in a language other than English, (6) articles without full-text availability, and (7) duplicated research (Table 1.)

### 2.3. Study Selection

The selection of studies was refined independently by two authors according to the predefined eligibility criteria (A.H./A.F.S.). The articles retrieved from the three electronic databases used were initially screened for inclusion based on the title of the article and the abstract. Thereafter, the full texts of potentially relevant studies were obtained and assessed by the same two authors (A.H./A.F.S.). Due to the paucity of relevant data, case series and reports were included. Duplicate publications were excluded. Discrepancies were resolved through discussion between the two reviewers and, when required, by consulting a third reviewer (I.J.).

### 2.4. Data Extraction

The study characteristics and results of the included studies were extracted by both reviewers. The following data variables were extracted for the observational and genetic studies: reference, country of study, study population and design, MTHFR variant, clinical outcomes, and thromboembolic event. For the case studies concerning MTHFR and COVID-19 thrombotic complications, the variables extracted were as follows: reference, year of publication, study population and design, presenting complaint, diagnosis/thromboembolic event, acquired risk factors, inherited risk factors, homocysteine level, treatment, and outcome. For case-studies with adverse outcomes post-vaccination and MTHFR mutations, the data extracted were as follows: reference, year of publication, country, study population and design, vaccine properties and manufacturer, presenting complaint, time since vaccination, adverse events, acquired risk factors, inherited risk factors, homocysteine level, treatment, and outcome.

### 2.5. Quality Assessment

To evaluate the quality of the included literature, appraisal checklists from the Joanna Briggs Institute (JBI) were employed.

## 3. Results

**Figure 1:**
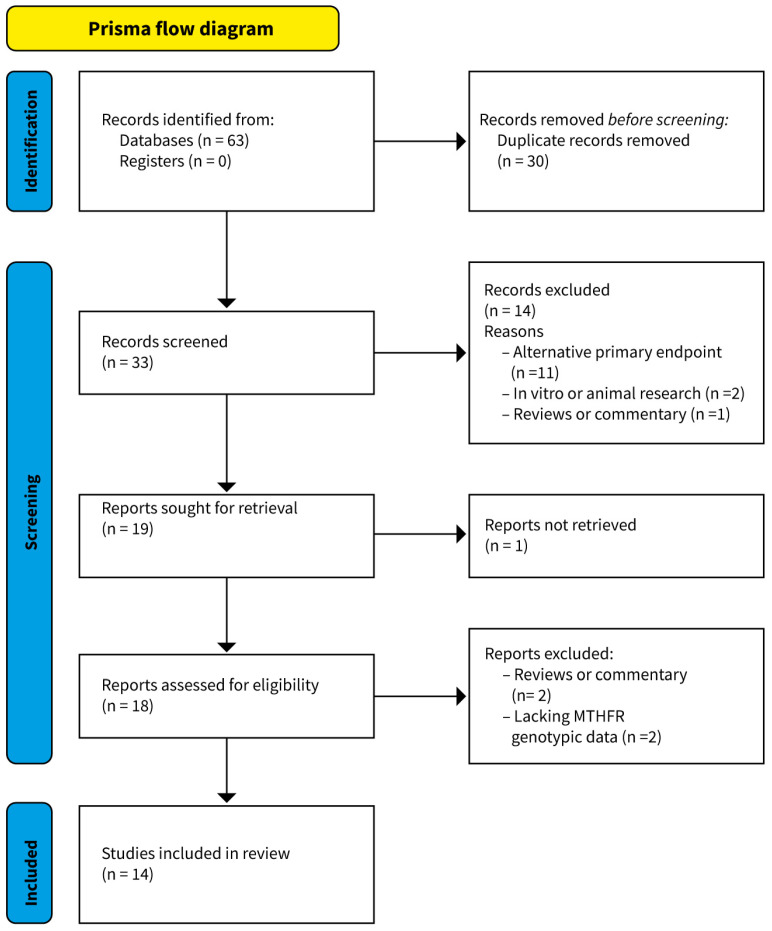
Preferred Reporting Items for Systematic Reviews and Meta-Analyses (PRISMA) flow diagram showing the selection of studies in this analysis.

The flow diagram of the database search and selection process is represented in Figure 1. The literature search identified 63 potentially relevant titles and abstracts, of which 24 were identified from the PubMed database search, 19 from Web of Science, and the remaining 20 from Scopus. A total of 33 records remained after 30 duplicates were identified and removed. Of these, a further 14 articles were excluded based on the initial screening of study titles and abstracts. A total of 18 full-text articles were retrieved and fully reviewed. Among them, four were excluded for reasons outlined in Figure 1. Finally, 14 studies met the full inclusion criteria for the systematic review.

Table 2 demonstrates the characteristics of the eight observational studies included in the review. As detailed, six of these studies were conducted in Europe (one in Croatia, two in Turkey, three in Italy). The remaining two studies were conducted in Asia (one in Russia, one in Uzbekistan). The thromboembolic events reported in the studies included pulmonary embolism, disseminated intravascular coagulation, acute cardiovascular events, myocardial infarction, acute cerebrovascular injury, venous thromboembolism, and thrombosis [15,16,17,18,19,20,21,22]. All studies included both male and female study participants and the most commonly occurring MTHFR polymorphisms (C677T and A1298C). Of the eight studies, four established an association between the presence of hereditary MTHFR variants and COVID-19 severity or specified thromboembolic events (*n* = 2200). The remaining four studies failed to identify a link between MTHFR and SARS-CoV-2 outcomes (*n* = 538) [15,16,17,18,19,20,21,22].

Ponti et al. conducted a retrospective observational study and concluded that there was a clear trend toward a connection between the worldwide prevalence of MTHFR 677 T and COVID-19 incidence and mortality. The population frequency of the C677 T allele showed regional and ethnic variations. The prevalence of the MTHFR 677 T allele in the Latino population, and the incidence and mortality for COVID-19 was higher for this ethnic group than that reported for most other populations globally. Statistical analysis showed a relatively strong correlation between C677 T and death from coronavirus. Ponti et al. concluded that genetic polymorphism of MTHFR C677 T may modulate the incidence and severity of COVID-19 pandemic infection [18].

Khidoyatovna et al. concluded that, according to their study conducted with the Uzbek population, the amount of homocysteine in the blood was significantly higher in patients with the non-wild-type allele of the MTHFR gene 677 C>T and 1298 A>C polymorphisms, compared with the control group, as well as to patients with the wild-type homozygous genotype. Similarly, it was found that almost all of the patients with T and C mutant alleles of the polymorphisms presented in the patients had moderate or severe disease. In addition to inflammation and coagulopathy induced by COVID-19, hyperhomocysteine exposure significantly increased the likelihood of severe COVID-19 disease compared with homozygous wild-type-genotype patients [19].

Cappadona et al. confirmed the contribution of the *MTHFR* variant chr1:11753033:G: A to the predisposition to severe COVID-19 infection [17].

Tekcan et al. concluded that MTHFR C677T CT genotype T allele was more prevalent in the intensive care of COVID-19-positive patients compared with other groups. Patients with PCR-positive results had a higher probability of the MTHFR C677T C/C genotype. In CT-positive patients, the MTHFR C677T CT genotype was more common and the MTHFR C677T variant affected the course of COVID-19 disease in the Turkish population [22].

Table 3 presents the findings of two case studies and one case report which identified COVID-19-associated thrombotic events in the presence of MTHFR mutations. One study comprised female participants only [23], two of whom were taking oestrogen-based oral contraceptives at the time of study. The remaining two studies consisted of male participants [24,25]. The age of patients ranged from 15 to 39 years. Homocysteine levels were elevated in only one of these studies [24]. The thromboembolic events recorded in the case studies were as follows: pulmonary embolism, infrarenal thrombosis, superior mesenteric thrombus, vena cava inferior thrombosis, ischemic stroke, aortic arch thrombus, complete right internal carotid artery occlusion, and acute kidney injury. All patients were treated with low-molecular-weight heparin.

In Table 4, we present one case series and two case reports with thromboembolic events as adverse effects of the COVID-19 vaccine. Three patients received mRNA-based vaccines, and one patient received a vector-based vaccine. Registered thromboembolic events were central venous sinus thrombosis, deep-vein thrombosis, and pulmonary embolism; however, only one death was registered.

## 4. Discussion

The results of this systematic review of the literature regarding the association between thromboembolic events in patients with COVID-19 infection and methylenetetrahydrofolate reductase (MTHFR) gene polymorphism demonstrated heterogenous results, although there were significant pieces of evidence that the MTHFR gene variants might be associated with thromboembolic complications of the COVID-19 infection. On the other hand, based only on case reports or case series, there were insufficient findings to conclude that vaccination against COVID-19 could be associated with thromboembolic incidents in patients with MTHFR gene polymorphism.

Out of a total of eight cross-sectional or observational studies, four studies indicated a connection between MTHFR variants and the outcome of COVID-19 infection, while the remaining four studies did not find a link between MTHFR variants (MTHFR C667T and MTHFR A1298C) and thromboembolic events and more severe outcomes of COVID-19 infection [15,16,17,18,19,20,21,22]. When we compared study population groups, the total number of subjects where the connection between the MTHFR gene variant and thromboembolic events was not proven was 538. In the study population groups where association between MTHFR variant and thrombosis was proven, the number of subjects was 2200, not including the subjects in the study of Ponti et al. [18], which was designed as a large retrospective observational genetic correlation population analysis. Thus, the number of subjects in studies with proven correlations between worse outcomes of COVID-19 infection (including thromboembolic events) and MTHFR variants was higher than the number of participants in studies where no such connections were found. Currently, there is an insufficient number of subjects to confirm a clear association between the MTHFR gene polymorphism and the morbidity and mortality of the COVID19 infection. Prospective and interventional studies with a large number of subjects are needed to confirm the strong association between the MTHFR variant and thromboembolic incidents and clinical outcomes of COVID-19 infection.

Furthermore, Ponti et al. [18] also concluded that a comparison of the prevalence of the homozygous *MTHFR*-677 mutation and the incidence of and mortality by COVID-19 showed a high degree of correlation and found geographical and populational divergences of this correlation, including Americans, Europeans, and Asians. A correlation between *MTHFR* 677 T prevalence and COVID-19 incidence and mortality rates could be observed if data were stratified for different ethnic groups, demonstrating the presence of a gradient along South–North and East–West directions worldwide. The frequency of the *MTHFR* C677 T allele in the Latino population (50%) was higher than that reported for most of the other populations in the world. In addition, coronavirus death correlation with the frequency of this allele in most of the different populations was lower (Finnish, African sub-Saharan, etc.). Correlation analysis showed a relatively strong correlation of 85% (*p* = 0.03) between C677 T and death caused by coronavirus. Therefore, Ponti et al. concluded that genetic polymorphism of *MTHFR* C677 T and homocysteine levels might modulate the risk of COVID-19 incidence, severity, and mortality. These data could be useful in defining better population-based risk strategies to fight COVID-19 infection and lethality [18].

Additionally, the genetic data related to MTHFR status coupled with homocysteine serum levels could represent important information for the assessment and stratification of COVID-19 patients and lethality prediction. It has been demonstrated that the presence of *MTHFR* T677 may result in the need for a higher folate and B-vitamin intake to maintain normal homocysteine levels. Therefore, a preventive therapeutic integration of folic acid and B vitamins could result in a reduction in the prevalence and mortality of COVID-19 viral infection [29]. On the other hand, in a cohort of 176 patients, Fevraleva et al. [21] could not demonstrate a higher risk of thrombotic complications during COVID-19 disease in carriers of the genetic markers for thrombophilia, including G1691A in the *FV* gene, C677T and A1298C in the *MTHFR* gene, G20210A in the *FII* gene, and (-675) 4G/5G in the *PAI-I* gene, relative to patients without these markers. Both groups were taking therapeutic doses of anticoagulants. As soon as anticoagulant therapy was applied, the presence of mutations in the genes of hereditary thrombophilia did not affect the severity of the course of COVID-19, the volume of lung damage according to CT scans, the patient’s intensive-care-unit stay, or hospital mortality [21]. This Croatian study included 30 severe COVID-19 patients (mean age: 61 years, from 33 to 86; 60% males) treated in the ICU and 49 non-severe COVID-19 patients (mean age: 57, from 26 to 88; 51% males). The association with an increased risk for severe COVID-19 was not found for the MTHFR C677T or MTHFR A1298C variants [21].

On the contrary, in a meta-analysis, Cappadona et al. confirmed the contribution of the *MTHFR* variant chr1:11753033: G: A to the predisposition to severe COVID-19. In addition to virus intrinsic characteristics, the host genetic makeup was predicted to account for the extreme clinical heterogeneity of the disease, which was characterized, among other manifestations, by a derangement of hemostasis associated with thromboembolic events. This analysis (Italian cohort = 2000 participants) confirmed the involvement of MTHFR gene polymorphism in predisposition to severe COVID-19 [15].

Results of a Turkish prospective observational study (*n* = 100) also confirmed the contribution of the MTHFR C677T CT genotype to COVID-19 morbidity and mortality.

The exact mechanisms by which COVID-19 infection influences the metabolism of homocysteine, and thereby increases the frequency of thromboembolic events, are not fully understood. It has recently been shown that SARS-CoV-2 remodels both the host folate and the one-carbon metabolism at the post-transcriptional level to meet the demand for viral subgenomic RNA replication, bypassing viral shutoff of host translation [30]. Since folate is depleted in SARS-CoV-2-infected cells, homocysteine levels should increase eventually leading to hyper-homocysteinemia, a known risk factor for a variety of complex disorders, including cardiovascular and neurological diseases, possibly contributing to a severe course of COVID-19 and thromboembolic incidents [7,31].

So far, only three publications regarding thromboembolic adverse effects after the COVID-19 vaccination have published incidents [26,27,28]. Three patients received mRNA-based vaccine and one patient received vector-based vaccine. Registered thromboembolic events were central venous sinus thrombosis, deep-vein thrombosis, and pulmonary embolism; however, only one death was registered. Thromboembolic complications occurred mainly after the first dose of the vaccine for COVID-19, in the period from 7 to 16 days after the first dose of the vaccine, and on the first day after two doses of the vaccine. Regarding COVID-19 vaccination and MTHFR mutations, the paucity of data (there are the only case reports, *n* = 4) precludes any conclusion regarding a possible association.

However, it is important to emphasize that our report had some limitations. Namely, the differences between the observed studies included in this review and their relatively small number were not sufficient to draw unequivocal conclusions. The studied populations and clinical outcomes, and the designs of the studies contained an extreme level of heterogeneity and were not comparable. Also, all included studies were observational or retrospective in design, or were only case reports or case series.

## 5. Conclusions

This systematic review article points out a possible association between the MTHFR gene variant and severe COVID-19 infection, especially thromboembolic outcomes. Although the results of studies included in this systematic review are conflicting due to their large heterogeneity, there is a growing body of evidence confirming the potential association between homozygotes of the MTHGR gene variant and more severe/thromboembolic outcomes of COVID-19 infection. The evidence for an association between the COVID-19 vaccines and thromboembolic events is insufficient for a firm conclusion. Further prospective multicentric trials with a larger number of subjects, longer follow-up, and additional outcomes, including determination of homocysteine plasma concentration, are needed to determine the connection between the MTHFR gene variant and COVID-19 infection or vaccination against SARS-CoV-2. Some of those trials have to be interventional studies investigating the possible role of B vitamins, including folic acid, in the treatment of COVID-19 and the prevention of thromboembolic events.

## Figures and Tables

**Table 1:** Inclusion and exclusion criteria.

Inclusion Criteria	Exclusion Criteria
Study type: Cross-sectional studies Cohort studies Case series Case studies Case reports	Study type: Narrative reviews Commentary
Studies evaluating MTHFR genotypes and COVID-19 severity in the context of thromboembolic events	Studies with primary endpoints outside the scope of our review (e.g., MTHFR polymorphisms and COVID-19-associated neuro-psychiatric conditions)
Studies reporting data on MTHFR polymorphisms and thrombotic events after COVID-19 vaccination	Studies lacking MTHFR genotypic data
Human studies	Animal and in vitro studies
	Research published in a language other than English
	Articles without full-text availability
	Duplicated research

**Table 2:** Characteristics of observational studies.

Reference, Country	Study Population and Design	MTHFR Variant	Clinical Outcomes	Thromboembolic Event	Conclusion
Lapic et al., 2022 [15], Croatia	M/F (*n* = 79), cross-sectional pilot study, two study subsets Severe COVID-19 (*n*= 30) Non-severe COVID-19 (*n* = 49)	C677T A1298C	Severe COVID-19, bilateral pneumonia, and at least one of the following: SpO_2_ < 94% PaO_2_/FiO_2_ < 300 mmhg RR > 30/min Lung infiltrates > 50% (requiring respiratory support)	Thrombosis DIC PE Acute CV event	No clear association between either MTHFR polymorphism and COVID-19 severity
Kose et al., 2023 [16], Turkey	M/F (*n* = 189), cross-sectional study, two study subsets Severe COVID-19 (*n* = 45) Non-severe COVID-19 (*n* = 144)	C677T A1298C	Severe COVID-19 SpO_2_ < 90% PaO_2_/FiO_2_ < 300 mmHg RR ≥ 30/min HR > 100 beats/min Hypotension Lactate > 2 mmol/L Dyspnoea Requiring > 5 L/min nasal O_2_ Renal dysfunction Confusion Thrombocytopenia Sepsis/septic shock Moderate/severe pneumonia Need for cytokine therapy or Abx	Assessed via increased D-dimer levels	No relationship between MTHFR variants and (1) D-dimer levels or (2) COVID-19 severity
Cappadonna et al., 2021 [17], Italy	M/F (*n* = 2000), genetic-association analyses, two study subsets Severe COVID-19 (*n* = 332) Control group (*n* = 1668)	MTHFR chr1:11753033: G: A (rs17875978)	Severe COVID-19 Defined as hospitalisation with respiratory failure	Not specified	Significant evidence of association between COVID-19 severity and MTHFR variant
Ponti et al., 2021 [18], Italy	M/F, retrospective observational genetic-correlation population analysis Latino European (non-Finnish) and Finnish East Asian South Asia Africa	C677T	COVID-19-related death	Not specified	Significant relationship between MTHFR C677T allele frequency in different populations and COVID-19 death
Khidoyatovna et al., 2022 [19], Uzbekistan	M/F *(n* = 100) Mild COVID-19 (*n* = 20) Moderate COVID-19 (*n* = 26) Severe COVID-19 (*n* = 34) Control group (*n* = 20)	C677T A1298C	Mild COVID-19 Moderate COVID-19 Severe COVID-19	Not specified	Positive association between MTHFR C677T and A1298C and the development of severe COVID-19
Fiorentino et al., 2023 [20], Italy	M/F (*n* = 94), retrospective observational genetic analysis, two study subsets Severe COVID-19 with PE (*n* = 47) Severe COVID-19 without PE (*n* = 47)	C677T A1298C	Severe COVID-19 RR > 30 bpm PaO_2_/FiO_2_ < 300 SaO_2_ 93% at rest Requiring mechanical ventilation	PE	No relationship between MTHFR C677T or A1298C and pulmonary embolism in COVID-19
Fevraleva et al., 2023 [21], Russia	M/F (*n* = 176), prospective observational study	C677T A1298C	Severe COVID-19 Volume of lung damage Requiring ICU Outcome of hospitalisation (recovery, death, transfer to another hospital)	VTE ACE MI PE	No relationship between MTHFR C677T or A1298C and thrombotic complications in COVID-19
Tekcan et al., 2023 [22], Turkey	M/F (*n* = 100), prospective observational study, 3 study subsets Requiring hospital admission Requiring ICU admission Outpatient care	C677T	Severe COVID-19 Requiring hospital admission Requiring ICU admission	Not specified	Significant evidence of association between MTHFR C677T variant and COVID-19 requiring ICU admission

Legend: M/F = male and female, PE = pulmonary embolism, DIC = disseminated intravascular coagulation, acute CV event = cardiovascular event, VTE = venous thromboembolism, ACE = acute cerebrovascular event, MI = myocardial infarction, ICU = intensive care unit.

**Table 3:** Characteristics of case studies with COVID-19, thromboembolic complications, and MTHFR mutations.

Reference, Country	Study Population and Design	Presenting Complaint	Thromboembolic Event	Acquired Risk Factors	Inherited Risk Factors	Homocysteine (μmol/L)	Treatment	Outcome
Saptoka et al., 2022 [23], USA	Females (*n* = 3), case series **P1** = 17 y **P2** = 17 y **P3** = 15 y	**P1** = chest pain, left lower extremity pain **P2** = abdominal pain, left shoulder pain **P3** = fever, RLQ pain	**P1** = DVT, bilateral PE **P2** = infra-renal IVC thrombus and PE **P3** = superior mesenteric thrombus	**P1** = OCP, COVID-19 ve+ IgG **P2** = OCP, COVID-19 ve+ IgG **P3** = COVID-19 +ve IgG	**P1** = homozygous *C677T* **P2** = heterozygous *C677T* and *A1298C* **P3** = heterozygous *C677T* and *A1298C*	**P1** = 8.9 **P2** = 6.5 **P3** = 8.9	**P1** = enoxaparin **P2** = enoxaparin **P3** = enoxaparin	**P1** = recovery **P2** = recovery **P3** = recovery
Tabatabaee et al., 2022 [24], Iran	Males (*n* = 2), case series **P1** = 39 y **P2** = 34 y	**P1** = fever, chills, malaise, cough **P2** = right hemiparesis and Broca’s aphasia	**P1** = ischemic stroke, aortic arch thrombus, complete RICA occlusion **P2** = ischemic stroke, secondary AKI	**P1** = PCR COVID-19 ve+ **P2** = PCR COVID-19 ve+	**P1** = homozygous *A1298C* **P2** = homozygous *C677T*	**P1** = 50 (↑) **P2** = 62 (↑)	**P1** = remdesivir, dexamethasone, clot retrieval with rTPA, DAPT and atorvastatin, heparin infusion, rivaroxaban **P2** = DAPT and atorvastatin, heparin, rivaroxaban, AKI, methylprednisone	**P1** = discharged, lost to follow-up **P2** = discharged, lost to follow-up
Staropoli et al., 2022 [25], USA	Male (*n* = 1), case report **P1** = 15 y	**P1** = painless blurry vision left eye	**P1** = central retinal vein occlusion (CRVO)	**P1** = PCR COVID-19 ve+	**P1** = homozygous MTHFR (variant unspecified)	**P1** = “normal”	**P1** = enoxaparin, intravitreal bevacizumab	**P1** = recovery

Legend: P1 = patient 1, P2 = patient 2, P3 = patient 3, RLQ = right lower quadrant, DVT = deep-vein thrombosis, RICA = right internal carotid artery, AKI = acute kidney injury, DAPT = dual anti-platelet therapy, ↑: increment.

**Table 4:** Characteristics of case studies with adverse outcomes post COVID-19 vaccination.

Reference, Country	Study Population and Design	Manufacturer, Vaccine Properties	Presenting Complaint	Time since Vaccination	Adverse Event	Acquired Risk Factors	Inherited Risk Factors	Homocysteine (μmol/L)	Treatment	Outcome
Fousse et al., 2022 [26], Germany	Females (*n* = 2), case series **Patient 1** = 20 y **Patient 2** = 28 y	AstraZeneca, vector-based	**P1** = drug refractory headache and unilateral calf pain **P2** = drug refractory headache	**P1** = 11 days (1st dose) **P2** = 16 days (1st dose)	**P1** = CVS **P2** = CVST	**P1** = OCP, ↑ b2 glycoprotein, Hashimoto’s **P2** = OCP, previous PE post-op	**P1** = heterozygous *A1298C* **P2** = homozygous *C677T* and heterozygous *PAI-1*	-	**P1** = LMWH and discontinue OCP **P2** = LMWH and discontinue OCP	**P1** = recovery **P2** = recovery
Atoui et al., 2022 [27], Lebanon	Male (*n* = 1), case report **Patient 1** = 24 y	Pfizer, mRNA-based	**P1** = pleuritic chest pain, low-grade fever and right lower extremity oedema, erythema, pain	**P1** = 1 day (2nd dose)	**P1** = DVT and PE	-	**P1** = homozygous MTHFR *A1298C*, heterozygous *FVL G1691A*	**P1** = 254 (↑)	**P1** = enoxaparin, apixaban (TTO)	**P1** = recovery
Franchini et al., 2022 [28], Italy	Male (*n* = 1), case report **Patient 1** = 50 y	AstraZeneca, mRNA-based	**P1** = worsening headache	**P1** = 7 days (1st dose)	**P1** = CVST	Positive FHx for thrombotic and haemorrhagic disorder	**P1** = heterozygous *C677T*	**P1** = 16.7 (↑)	**P1** = neurosurgery	**P1** = death

Legend: CVST = central venous sinus thrombosis, OCP = oral contraceptive pill (estrogen-containing), PAI-1 = prothrombin activator inhibitor-1, FVL G1691A = Factor V Leiden mutation, DVT = deep-vein thrombosis, PE = pulmonary embolism, FHx = family history, TTO = to take out (discharged with). ↑: increment.

## Data Availability

The data presented in this study are available on request from the corresponding author.

## References

[1] Song C., Li Z., Li C., Huang M., Liu J., Fang Q., Cao Z., Zhang L., Gao P., Nie W. (2022). SARS-CoV-2: The Monster Causes COVID-19. Front. Cell. Infect. Microbiol..

[2] COVID Live—Coronavirus Statistics—Worldometer. https://www.worldometers.info/coronavirus/.

[3] Abu-Farha M., Al-Sabah S., Hammad M.M., Hebbar P., Channanath A.M., John S.E., Taher I., Almaeen A., Ghazy A., Mohammad A. (2020). Prognostic Genetic Markers for Thrombosis in COVID-19 Patients: A Focused Analysis on D-Dimer, Homocysteine and Thromboembolism. Front. Pharmacol..

[4] Sui J., Noubouossie D.F., Gandotra S., Cao L. (2021). Elevated Plasma Fibrinogen Is Associated with Excessive Inflammation and Disease Severity in COVID-19 Patients. Front. Cell. Infect. Microbiol..

[5] Abou-Ismail M.Y., Diamond A., Kapoor S., Arafah Y., Nayak L. (2020). The hypercoagulable state in COVID-19: Incidence, pathophysiology, and management. Thromb. Res..

[6] Parasher A. (2020). COVID-19: Current understanding of its Pathophysiology, Clinical presentation and Treatment. Heart.

[7] Karst M., Hollenhorst J., Achenbach J. (2020). Life-threatening course in coronavirus disease 2019 (COVID-19): Is there a link to methylenetetrahydrofolic acid reductase (MTHFR) polymorphism and hyperhomocysteinemia?. Med. Hypotheses.

[8] Liew S.-C., Das Gupta E. (2015). Methylenetetrahydrofolate reductase (MTHFR) C677T polymorphism: Epidemiology, metabolism and the associated diseases. Eur. J. Med. Genet..

[9] Moll S., Varga E.A. (2015). Homocysteine and *MTHFR* Mutations. Circulation.

[10] Aday A.W., Duran E.K., Van Denburgh M., Kim E., Christen W.G., Manson J.E., Ridker P.M., Pradhan A.D. (2021). Homocysteine Is Associated with Future Venous Thromboembolism in 2 Prospective Cohorts of Women. Arter. Thromb. Vasc. Biol..

[11] Carpenè G., Negrini D., Henry B.M., Montagnana M., Lippi G. (2022). Homocysteine in coronavirus disease (COVID-19): A systematic literature review. Diagnosis.

[12] Keskin A., Ustun G.U., Aci R., Duran U. (2022). Homocysteine as a marker for predicting disease severity in patients with COVID-19. Biomark. Med..

[13] Gadiyaram V.K., Khan M.A., Hogan T., Altaha R., Crowell E., Hobbs G., Perrota P. (2009). Significance of MTHFR gene mutation with normal homocysteine level in vascular events. J. Clin. Oncol..

[14] Moher D., Liberati A., Tetzlaff J., Altman D.G. (2010). Preferred reporting items for systematic reviews and meta-analyses: The PRISMA statement. Int. J. Surg..

[15] Lapić I., Antolic M.R., Horvat I., Premužić V., Palić J., Rogić D., Zadro R. (2022). Association of polymorphisms in genes encoding prothrombotic and cardiovascular risk factors with disease severity in COVID-19 patients: A pilot study. J. Med. Virol..

[16] Kose M., Senkal N., Konyaoglu H., Emet A., Oyaci Y., Pehlivan S., Sayin G.Y. (2023). The effect of hereditary thrombotic factors and comorbidities on the severity of COVID-19 disease. Eur. Rev. Med. Pharmacol. Sci..

[17] Cappadona C., Paraboschi E.M., Ziliotto N., Bottaro S., Rimoldi V., Gerussi A., Azimonti A., Brenna D., Brunati A., Cameroni C. (2021). MEDTEC Students against Coronavirus: Investigating the Role of Hemostatic Genes in the Predisposition to COVID-19 Severity. J. Pers. Med..

[18] Ponti G., Pastorino L., Manfredini M., Ozben T., Oliva G., Kaleci S., Iannella R., Tomasi A. (2021). COVID-19 spreading across world correlates with C677T allele of the methylenetetrahydrofolate reductase (MTHFR) gene prevalence. J. Clin. Lab. Anal..

[19] Khidoyatovna I.F., Agzamovna B.S., Chutbaevna K.Z. (2022). Relationship between MTHFR gene RS1801133 and rs1801131 polymorphisms with disease severity of COVID-19 and Homocystein levels in Uzbek patients. J. Pharm. Negat. Results.

[20] Fiorentino G., Benincasa G., Coppola A., Franzese M., Annunziata A., Affinito O., Viglietti M., Napoli C. (2022). Targeted genetic analysis unveils novel associations between ACE I/D and APO T158C polymorphisms with D-dimer levels in severe COVID-19 patients with pulmonary embolism. J. Thromb. Thrombolysis.

[21] Fevraleva I., Mamchich D., Vinogradov D., Chabaeva Y., Kulikov S., Makarik T., Margaryan V., Manasyan G., Novikova V., Rachina S. (2023). Role of Genetic Thrombophilia Markers in Thrombosis Events in Elderly Patients with COVID-19. Genes.

[22] Tekcan A., Cihangiroglu M., Capraz M., Capraz A., Yigit S., Nursal A.F., Menekse E., Durmaz Z.H., Demir H.D., Ozcelik B. (2023). Association of *ACE* ID, *MTHFR* C677T, and *MIF*-173GC variants with the clinical course of COVID-19 patients. Nucleosides Nucleotides Nucleic Acids.

[23] Sapkota B., Karroum S.B., Dillawn S., Daines B., Zaid-Kaylani S., Parat S., Bhaskaran S. (2022). Does COVID-19 Infection Increase the Risk of Hypercoagulability in Individuals with MTHFR Gene Mutation?. J. Med. Case Rep. Case Ser..

[24] Staropoli P.C., Payson A., Negron C.I., Prakhunhungsit S., Laufer P., Berrocal A.M. (2022). CRVO associated with COVID-19 and MTHFR mutation in a 15-year-old male. Am. J. Ophthalmol. Case Rep..

[25] Tabatabaee S., Rezania F., Soleimani S., Mirzaasgari Z. (2022). Hyperhomocysteinemia is related to large vessel occlusion in young patients with COVID -19: Two case reports. Clin. Case Rep..

[26] Fousse M., Schub D., Merzou F., Fassbender K., Sester M., Kettner M., Lochner P., Schmidt T., Júnior J.R.G. (2021). Case report: Cerebral sinus vein thrombosis in two patients with AstraZeneca SARS-CoV-2 vaccination. J. Neurol..

[27] Atoui A., Jarrah K., Al Mahmasani L., Bou-Fakhredin R., Taher A.T. (2022). Deep venous thrombosis and pulmonary embolism after COVID-19 mRNA vaccination. Ann. Hematol..

[28] Franchini M., Testa S., Pezzo M., Glingani C., Caruso B., Terenziani I., Pognani C., Bellometti S.A., Castelli G. (2021). Cerebral venous thrombosis and thrombocytopenia post-COVID-19 vaccination. Thromb. Res..

[29] Ibrahimagic O.C., Smajlovic D., Dostovic Z., Pasic Z., Kunic S., Iljazovic A., Hajdarevic D.S. (2016). Hyperhomocysteinemia and Its Treatment in Patients with Parkinson’s Disease. Mater. Socio Medica.

[30] Zhang Y., Guo R., Kim S.H., Shah H., Zhang S., Liang J.H., Fang Y., Gentili M., Leary C.N.O., Elledge S.J. (2021). SARS-CoV-2 hijacks folate and one-carbon metabolism for viral replication. Nat. Commun..

[31] Sharma P., Senthilkumar R., Brahmachari V., Sundaramoorthy E., Mahajan A., Sharma A., Sengupta S. (2006). Mining literature for a comprehensive pathway analysis: A case study for retrieval of homocysteine related genes for genetic and epigenetic studies. Lipids Heal. Dis..

